# Association between weight-adjusted-waist index and gallstones: an analysis of the National Health and Nutrition Examination Survey

**DOI:** 10.1186/s12876-024-03127-9

**Published:** 2024-01-18

**Authors:** Si-Hua Wen, Xin Tang, Tao Tang, Zheng-Rong Ye

**Affiliations:** 1 Department of Abdominal Surgery, The Third People’s Hospital of Yongzhou, Yongzhou, China; 2 Department of Hepatobiliary Surgery, The Central Hospital of Yongzhou, Yongzhou, China; 3 Department of Endocrinology, The Third People’s Hospital of Yongzhou, Yongzhou, China

**Keywords:** Weight-adjusted-waist index, Gallstones, Visceral fat, Cross-sectional study, NHANES

## Abstract

**Background:**

The weight-adjusted-waist index (WWI) is a novel obesity index, and gallstones are associated with obesity. This study aimed to investigate the possible relationship between WWI and gallstones.

**Methods:**

The datasets from the National Health and Nutrition Examination Survey (NHANES) 2017–2020 were used in a cross-sectional investigation. Multivariate linear regression models were used to examine the linear connection between WWI and gallstones incidence. Fitted smoothing curves and threshold effect analysis were used to describe the nonlinear relationship.

**Results:**

The study comprised 8004 participants over the age of 20, including 833 reported with gallstones. Participants in the higher WWI tertile tended to have a higher gallstones prevalence. In the final adjusted model, a positive association between WWI and gallstones prevalence was observed (OR = 1.34, 95% CI: 1.20‒1.49). Participants in the highest WWI tertile had a significantly 71% higher risk of gallstones than those in the lowest WWI tertile (OR = 1.71, 95% CI: 1.35‒2.17). A nonlinear correlation was found between the WWI and gallstones prevalence, with an inflection point of 12.7.

**Conclusions:**

Our study found that higher WWI levels connected with increased prevalence of gallstones. However, more prospective studies are needed to validate our findings.

## Introduction

Gallstones are one of the most prevalent gastrointestinal diseases and a common cause of hospitalization [1]. It affects approximately 10–20% of the adult population worldwide [2, 3]. Approximately 70% of patients with gallstones are asymptomatic and detected incidentally when a medical examination is performed. In between 3% and 8% of patients with gallstones, complications such as cholecystitis, pancreatitis, cholangitis, and gallbladder perforation might develop [4]. The formation of gallstones may be influenced by a combination of genetic and environmental factors, and in Western countries, 75–80% of gallstones are composed mainly of cholesterol [5]. In patients with symptomatic gallstones, cholesterol was detected in 95% of gallstones specimens [6]. In addition, gallstones pose a serious threat to developing gallbladder cancer, with a relative risk of 4.9 being present in 65–90% of individuals with the disease [7, 8]. Each year, more than a million Americans receive a new diagnosis of gallstones, and approximately 700,000 of these patients undergo cholecystectomy [2]. The economic costs associated with the disease are estimated at $6.5 billion annually, and the associated costs have been increasing rapidly [8]. Therefore, effective clinical indicators are valuable for predicting or preventing the development of gallstones.

Obesity is a global health problem. Growing obesity rates place a substantial financial and health burden on all nations, as it has also significantly increased the prevalence of cardiovascular disease, cancer and diabetes [9]. Abdominal obesity has been shown to be linked to insulin resistance (IR), possibly due to the release of more unstable fatty acids, increased movement of fatty acids to the liver, decreased circulating adiponectin levels, and accumulation of inflammatory cells [10], whereas subcutaneous adipose tissue is not directly related to IR [11]. IR raises triglyceride levels while lowering high-density lipoprotein (HDL) cholesterol levels, both of which increase the chance of developing gallstones [12–14]. The prevalence of cholesterol gallstones is sharply increasing, in parallel with the global epidemics of insulin resistance, diabetes, elevated levels of visceral fat, obesity, and metabolic syndrome [15]. Obesity, particularly abdominal obesity, may contribute to the formation of gallstone [16]. Previous research showed that increased visceral fat is connected to the development of gallstone and may result in the need for gallbladder surgery at a younger age [17]. In obese populations, cholesterol is over-secreted from the liver into the bile due to up-regulation of 3-hydroxy-3-methylglutaryl (HMG)-CoA reductase activity, which induces the precipitation and crystallization of cholesterol—onset of gallstones formation [18, 19].

More studies have recently highlighted the connection between metabolic disorders and abdominal obesity rather than merely individual overweight [20–22]. Several methods for assessing body fat distribution are available, including computed tomography (CT), magnetic resonance imaging (MRI) and magnetic resonance spectroscopy (MRS). However, the utilization of these techniques in clinical settings is constrained due to their drawbacks of being pricy, difficult to obtain, time-consuming, or radiated. Traditional anthropometric indices, such as body mass index (BMI) and waist circumference (WC), are insufficiently precise to evaluate the proportion of body composition, i.e., they do not reflect the percentage of visceral or body fat. Weight-adjusted-waist index (WWI) is a novel obesity index, calculated by standardizing WC for body weight, reducing the correlation with BMI and making it easy to measure. WWI reflects the fat-to-muscle composition ratio and fits across different grades of BMI [23]. WWI is highly correlated with obesity-related metabolic syndrome, and the use of WWI to predict gallstones development shows potential; however, the relationship between WWI and gallstones remains uncertain. Therefore, the aim of this study was to assess the relationship between WWI and gallstones in a massive, representative nationwide sample of U.S. adults. The dataset was obtained from the National Health and Nutrition Examination Survey (NHANES).

## Methods

### Survey description

The authors obtained data from NHANES (www.cdc.gov/nchs/nhanes), a national population-based cross-sectional study conducted by the National Center for Health Statistics (NCHS) to investigate health status in America; therefore, the sample was representative. All NHANES study protocols were approved by the Ethics Review Board, and all survey participants included in this study signed written informed consent.

### Study population

We collected data from 2017 to 2020 as only that cycle included participants who answered whether they had gallstone or not.

**Fig. 1:**
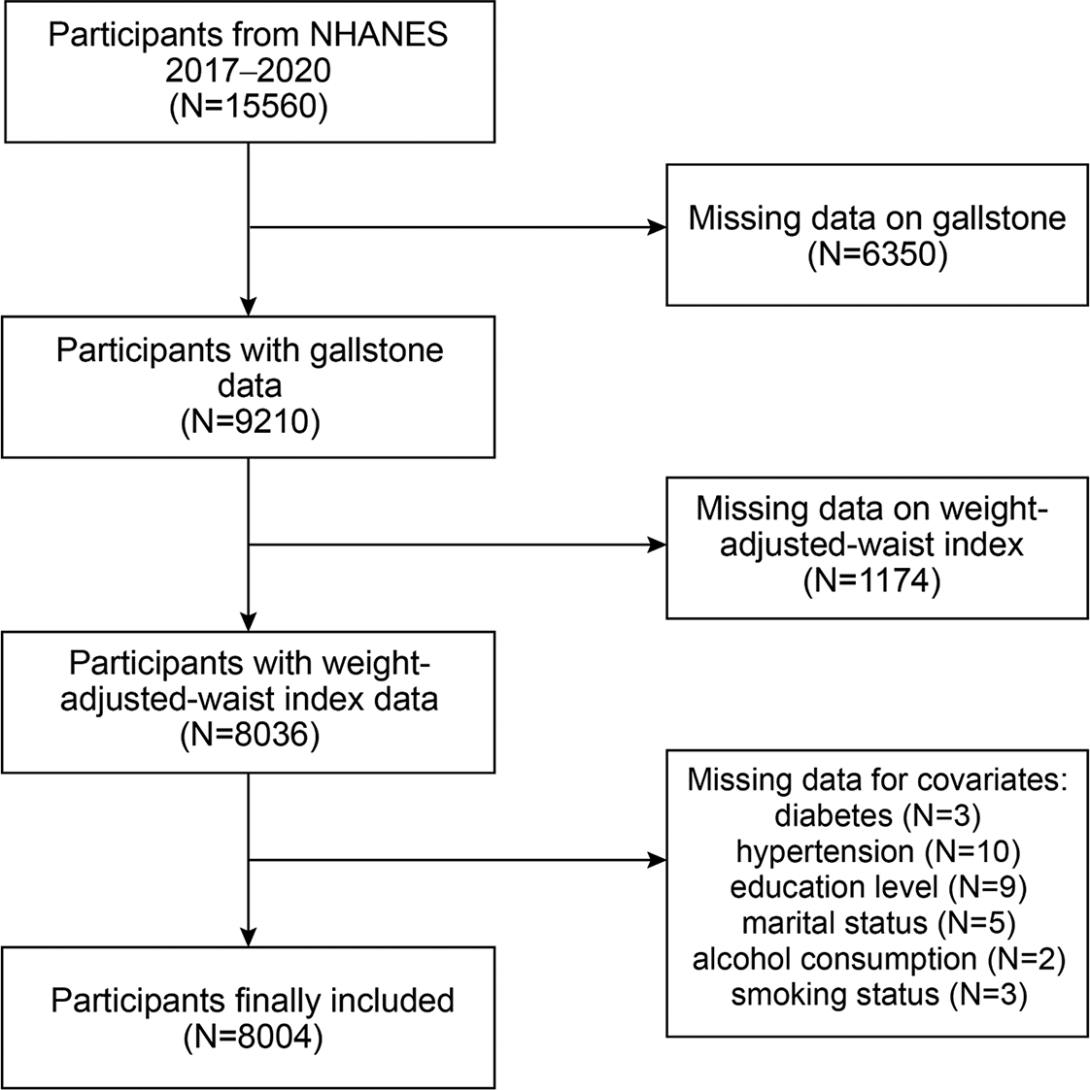
Flowchart of the sample selection from NHANES 2017–2020

A total of 15,560 participants were involved during the survey period, of which participants that contained information on gallstone (*n* = 9210) were included in this analysis. Those participants with missing WWI data were excluded (*n* = 1174). Missing data for covariates were also excluded (total, *n* = 32; diabetes, *n* = 3; hypertension, *n* = 10; education level, *n* = 9; marital status, *n* = 5; alcohol consumption, *n* = 2; smoking status, *n* = 3). The study eventually included 8004 participants (Fig. 1).

### Study variables

We used WWI as an exposure variable in our analysis. WWI is an obesity index based on WC, body weight, for assessment of abdominal obesity. The WWI was calculated as WC in centimeters divided by the square root of weight in kilograms.

In the present analysis, the identification of gallstone cases was conducted through questionnaires during the NHANES 2017–2020 survey. Referring to the interview guidelines of NHANES, participants were asked by trained interviewers as the following question “Have you ever had gallstones?”. If the participant had not previously consulted a physician or health professional, this condition was also treated as missing data. This classification method has been utilized in prior published studies as well [17].

### Covariates

The covariate data in our study were obtained from the NHANES official website. Continuous variables include age, dietary intake, income-to-poverty ratio (PIR), sedentary time (min), and triglyceride (mg/dL). Categorical variables include gender, race, education level, marital status (cohabitation/solitude), alcohol consumption, diabetes, hypertension, and smoking status. Dietary information included energy (kcal), sugar (g), fat (g), water (g), dietary fiber (g) and caffeine consumption (g). Diabetes and hypertension were defined based on participants’ responses to the questions: “Has a doctor ever told you that you have diabetes?” and “Has a doctor ever told you that you have high blood pressure?”

The marital status category included cohabitation (married/living with partner) and solitude (widowed/divorced/separated/unmarried). Participants who consumed alcohol at least once a month were considered drinkers, while those who had smoked at least 100 cigarettes in their lifetime were classified as smokers.

Dietary information was extracted from a 24-h dietary questionnaire, which included the mean of the sum of the participant-specific nutrient intakes for the 1st and 2nd 24-h periods was ultimately included in this study. Dietary data were transformed into categorical variables, with the 50th percentile of the sample size as the cut-off threshold, dividing them into “low” and “high” categories. Missing data in the covariates were categorized as “unclear”.

### Statistical analysis

Statistical analysis was performed using EmpowerStats 4.0. Continuous variables are summarized as mean ± SD, whereas categorical variables are expressed as proportions. Multiple logistic regression models were used to study the relationship between variables. The multivariate test was built using three models: In model 1, no covariates were adjusted; Model 2 was adjusted for gender, age, race, marital status, PIR, and education level; model 3: adjusted for all covariates. Fitted smoothing curves were performed by adjusting the variables. Using a threshold effects analysis model, we analyzed the link and inflection points between WWI and gallstones prevalence. Multiple logistic regression models were used to test the stability of the relationship across subgroups of gender, age, hypertension, diabetes, and sedentary time. We converted the variable “sedentary time” into a categorical variable, dividing it into three groups based on sample size. *P* < 0.05 was considered statistically significant.

## Results

### Baseline characteristics of participants

A total of 8004 participants were included and the baseline characteristics of the participants are shown below (Table 1). WWI was 11.56 (10.80, 12.32) in the group of participants with gallstones, compared to 11.07 (10.21, 11.93) in the group without gallstones (*P* < 0.001). There were no statistically significant differences within PIR, education level, marital status, sugar intake, and caffeine consumption (all *P* > 0.05).

**Table 1:** Baseline characteristics of participants

Characteristic	Non-stone formers (*n* = 7171)	Stone formers (*n* = 833)	P-value
Age (years)	49.75 ± 17.36	57.84 ± 15.8	< 0.001
Gender, n (%)			< 0.001
Male	51.18	29.05	
Female	48.82	70.95	
Race, n (%)			< 0.001
Mexican American	11.49	12.85	
Other Race	27.36	24.13	
Non-Hispanic White	33.80	42.38	
Non- Hispanic Black	27.35	20.65	
Education lever, n (%)			0.380
Less than high school	18.32	16.93	
High school	24.15	26.05	
More than high school	57.52	57.02	
Marital status, n (%)			0.449
Cohabitation	41.82	40.46	
Solitude	58.18	59.54	
PIR	2.62 ± 1.52	2.56 ± 1.46	0.344
Triglyceride (mg/dL)	109.32 ± 64.88	115.77 ± 66.06	0.007
Sedentary time (min)	328.00 ± 199.49	353.03 ± 206.52	< 0.001
Alcohol consumption, n (%)			< 0.001
Yes	40.96	52.22	
No	45.75	34.09	
Unclear	13.29	13.69	
Smoked, n (%)			0.007
Yes	58.76	53.90	
No	41.24	46.10	
Diabetic, n (%)			< 0.001
Yes	83.32	71.91	
No	16.68	28.09	
Hypertension, n (%)			< 0.001
Yes	63.76	45.26	
No	36.24	54.74	
Total Energy, n (%)			< 0.001
Lower (< 1915.5 kcal)	39.21	47.18	
Higher (≥ 1915.5 kcal)	40.55	35.65	
Unclear	20.23	17.17	
Total Sugar, n (%)			0.079
Lower (< 88.3 g)	40.02	40.22	
Higher (≥ 88.3 g)	39.74	42.62	
Unclear	20.23	17.17	
Total Fat, n (%)			0.014
Lower (< 77 g)	39.53	44.42	
Higher (≥ 77 g)	40.23	38.42	
Unclear	20.23	17.17	
Total Water, n (%)			0.005
Lower (< 2523.5 g)	39.46	45.02	
Higher (≥ 2523.5 g)	40.30	37.82	
Unclear	20.23	17.17	
Total Fiber, n (%)			0.004
Lower (< 14.5 g)	39.45	45.14	
Higher (≥ 14.5 g)	40.32	37.70	
Unclear	20.23	17.17	
Caffeine consumption, n (%)			0.110
Lower (< 93 g)	39.90	41.30	
Higher (≥ 93 g)	39.87	41.54	
Unclear	20.23	17.17	
WII	11.07 ± 0.86	11.56 ± 0.76	< 0.001

Mean ± SD for continuous variables: *P* value was calculated by weighted linear regression model

N(%) for categorical variables: *P* value was calculated by weighted chi-square test

PIR, income-to-poverty ratio; WWI, weight-adjusted-waist index

### Association between WWI and gallstones prevalence

Table 2 shows the association between WWI and gallstones prevalence. In the unadjusted model, a higher WWI was related to increased prevalence of gallstones (OR = 1.98, 95% CI:1.81–2.17). Likewise, in the final adjusted model (model 3), a steady link between WWI and gallstones was maintained (OR = 1.34, 95% CI:1.20–1.49), showing that the incidence of gallstones increased by 34% for every unit rises in WWI. Furthermore, we converted WWI to a trichotomies variable for sensitivity analysis and discovered a tendency showing that higher-level WWI (≥ 11.50) groups were more likely to get gallstones. (OR = 1.71, 95% CI:1.35‒2.17).

**Table 2:** Logistic regression analysis between WII and gallstone prevalence

WWI groups	Model 1 OR (95% CI)	Model 2 OR (95% CI)	Model 3 OR (95% CI)
**Continuous**	1.98 (1.81,2.17)	1.49 (1.34,1.65)	1.34 (1.20,1.49)
**Categories**			
Tertile 1 (< 10.77)	1.0	1.0	1.0
Tertile 2 (≥ 10.77, < 11.50)	2.11 (1.70,2.62)	1.55 (1.24,1.95)	1.40 (1.11,1.76)
Tertile 3 (≥ 11.50)	3.89 (3.17,4.76)	2.13 (1.69,2.67)	1.71 (1.35,2.17)
***P *** **for trend**	2.20 (1.96,2.46)	1.54 (1.35,1.75)	1.35 (1.19,1.55)

Model 1: no covariates were adjusted

Model 2: age, gender, race, income-to-poverty ratio, marital status, and education level were adjusted

Model 3: gender, age, race, education level, marital status, income-to-poverty ratio, caffeine consumption, alcohol consumption, triglyceride, hypertension, diabetes, smoking status, sedentary time, energy intake, fat intake, sugar intake, water intake, and dietary fiber were adjusted

**Fig. 2:**
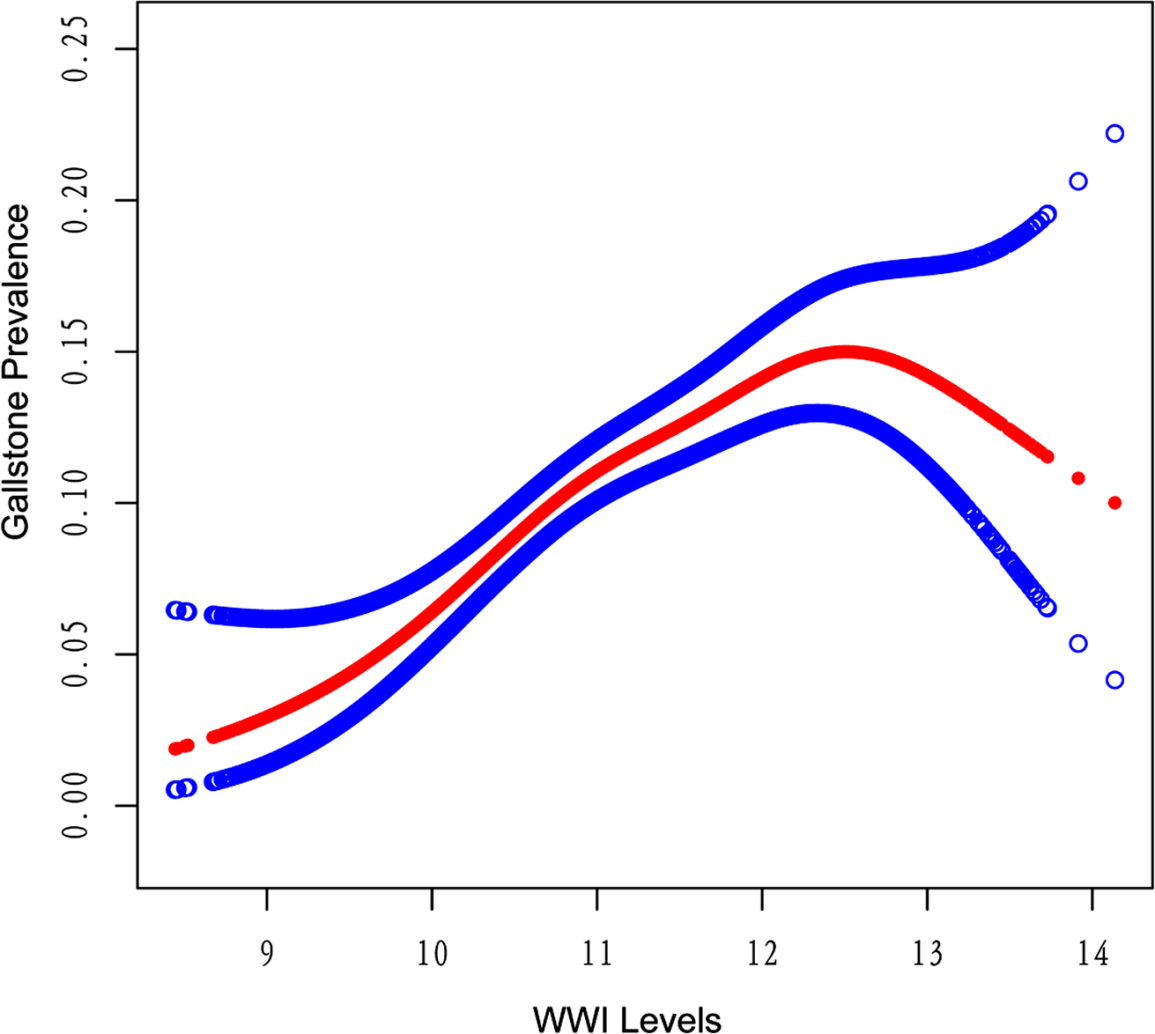
Density dose-response relationship between WWI with gallstone prevalence. 95% confidence interval (Cl) is displayed for the region between the upper and lower dashed lines. Adjusting for all covariates

Using a threshold effects analysis model, a nonlinear correlation was found between the WWI and gallstones prevalence (Fig. 2). There was a significant positive correlation between WWI and gallstones (OR = 1.45, 95% CI:1.29–1.63) to the left of the inflection point (12.7), and a significant negative correlation (OR = 0.39, 95% CI:0.17–0.64) to the right of the inflection point. (Loglikelihood ratio test, *P* = 0.002) (Table 3).

**Table 3:** Two-piecewise linear regression explained the threshold effect analysis of WII with gallstone prevalence

WWI	ULR Test	PLR Test	LRT Test
	OR (95% CI)	OR (95% CI)	OR (95% CI)
< 12.7, (*n* = 7761)	1.34 (1.20,1.49)	1.45 (1.29,1.63)	0.002
≥ 12.7, (*n* = 243)		0.39 (0.17,0.90)	

ULR, univariate linear regression; PLR, piecewise linear regression; LRT, logarithmic likelihood ratio test, statistically significant: *P* = 0.002

### Subgroup analysis

**Fig. 3:**
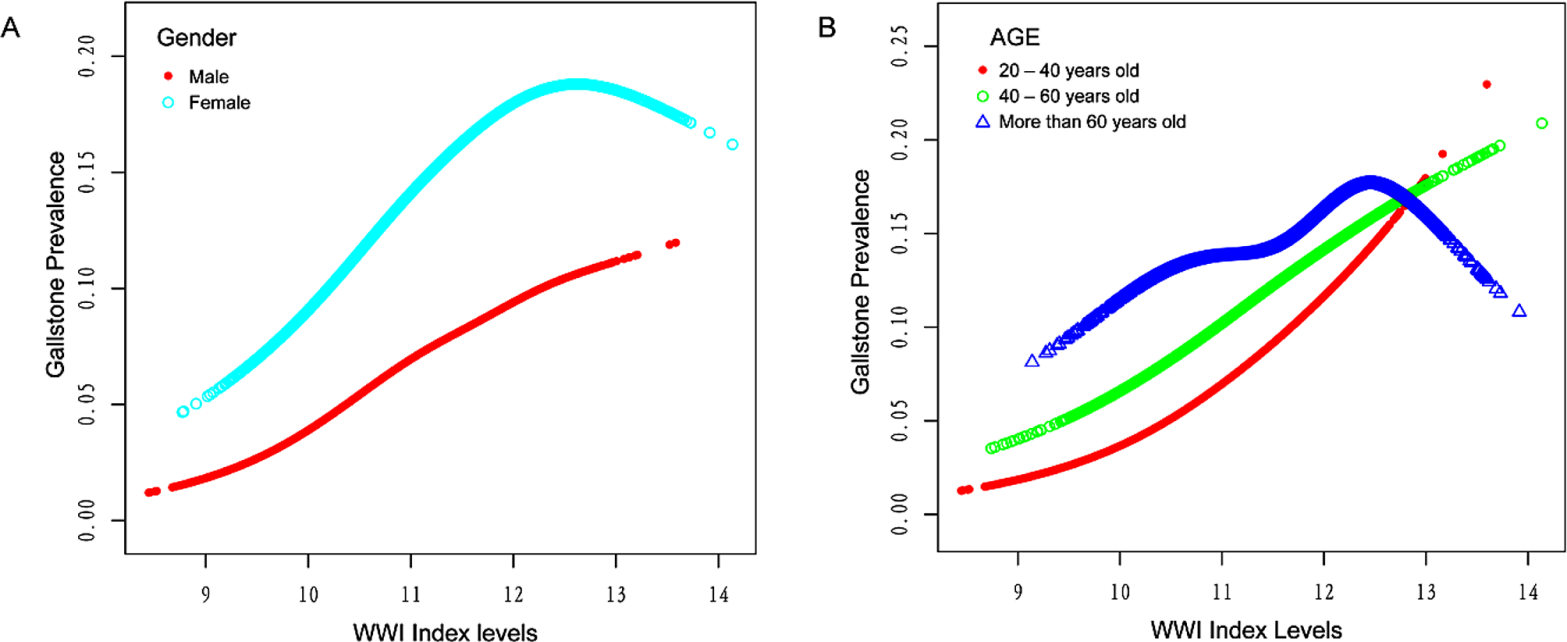
The association between WWl and gallstone prevalence stratified by (**A**) gender and (**B**) age

Subgroup analyses were used to evaluate the effect of WWI on gallstones and their stability in different populations (Table 4). There was still a significant positive association between WWI and gallstones in subgroups based on a number of variables, including gender, diabetes, hypertension, and sedentary time (*P* < 0.05). However, there was no significant difference in the association between WWI and gallstones prevalence in the subgroup “age ≥ 60 years” (*P* > 0.05). Subsequently, we presented fitted smoothing curves stratified by “age, gender”. In the stratified analysis, we found the similar inverted U-shaped curves in two populations including age > 60 years and female. The prevalence of gallstones was consistently higher in female participants than in male at the same WWI level (Fig. 3).

**Table 4:** Subgroup analysis between WWI with gallstone prevalence

Characteristic	Model 1 OR (95% CI)	Model 2 OR (95% CI)	Model 3 OR (95% CI)	P for interaction
**Stratified by gender**				0.2247
Male	2.16 (1.81,2.58)	1.68 (1.39,2.03)	1.48 (1.22,1.80)	
Female	1.69 (1.52,1.88)	1.43 (1.27,1.60)	1.30 (1.15,1.46)	
**Stratified by age (years)**				0.0017
< 40	2.35 (1.90,2.90)	2.01 (1.62,2.49)	1.83 (1.47,2.28)	
≥ 40, < 60	1.89 (1.61,2.22)	1.66 (1.41,1.96)	1.45 (1.22,1.72)	
≥ 60	1.52 (1.32,1.75)	1.29 (1.11,1.49)	1.15 (0.99,1.34)	
**Stratified by diabetes**				0.4733
Yes	1.78 (1.45,2.18)	1.33 (1.07,1.64)	1.25 (1.01,1.55)	
No	1.94 (1.75,2.14)	1.46 (1.30,1.64)	1.36 (1.21,1.53)	
**Stratified by hypertension**				0.0209
Yes	1.64 (1.44,1.87)	1.27 (1.10,1.47)	1.19 (1.03,1.38)	
No	2.05 (1.80,2.33)	1.59 (1.39,1.83)	1.49 (1.29, 1.72)	
**Stratified by sedentary time (min)**				0.1541
< 210	1.70 (1.44,2.00)	1.28 (1.07,1.52)	1.20 (1.00,1.44)	
≥ 210, < 330	1.92 (1.60,2.29)	1.40 (1.16,1.69)	1.28 (1.06,1.55)	
≥ 330	2.19 (1.92,2.50)	1.64 (1.42,1.89)	1.47 (1.27,1.17)	

Gender, age, race, education level, marital status, income-to-poverty ratio, caffeine consumption, alcohol consumption, triglyceride, hypertension, diabetes, smoking status, sedentary time, energy intake, fat intake, sugar intake, water intake, and dietary fiber were adjusted

In addition, we found that the positive association between WWI and the prevalence of gallstones was not significantly affected by stratifications, including gender, diabetes and sedentary time (all *P* for interaction > 0.05). Although interaction test for variable hypertension was statistically different (*P* < 0.05), both the population with and without hypertension continued to show the consistent positive connection.

## Discussion

In the cross-sectional study included 8004 participants, a positive correlation between WWI and gallstones incidence was found, with the incidence of gallstones increased by 34% for every unit rises in WWI (OR = 1.34, 95% CI:1.20–1.49). Meanwhile, there was no significant dependence of gender, diabetes, sedentary activity, or hypertension on the correlation, suggesting that higher levels of WWI may contribute to a higher prevalence of gallstones. Notably, as is shown in the fitted smoothing curves, we observed a nonlinear correlation between WWI and gallstones in the entire study population, with an inflection point at 12.7. The results showed a positive correlation between WWI and the prevalence of gallstones to the left of the inflection point, with the risk of prevalence approaching its peak when WWI was equal to 12.7. Based on these results, the management of WWI may attenuate the occurrence of gallstones.

To the best of our knowledge, this is the first study to investigate WWI and gallstones. Previous studies have shown the impact of WWI on a range of health conditions, including urinary protein excretion, all-cause mortality, and heart failure [24–26]. In 2018, Park et al. proposed that WWI was positively linked with the risk of cardiovascular metabolic disease and all-cause mortality; and these risks presented an inverse J-shaped pattern with waist-to-height ratio (WHtR), WC, and BMI [27]. BMI is generally restricted to measuring fat mass and is not sensitive to measuring fat distribution, especially when it is below 30 kg/m^2^ [28]. Various studies have shown the impact of visceral fat on metabolic syndrome, with even those who are at a healthy weight but with excess visceral adipose tissue are under significant risk of developing metabolic diseases [29–31]. However, an ultrasonography survey of a population in Mexico revealed that WHtR was not linked to gallstones [32]. Radmard et al. showed no significant association between subcutaneous fat and the development of gallstones [33]. Such evidence implied that the prevalence of gallstones in the clinical context may not be accurately reflected by conventional anthropometric markers. A cohort study of 1946 subjects underwent CT scans to measure abdominal fat and found that WWI had a positive association with visceral fat area and a negative association with abdominal muscle mass [34]. A two-year prospective study revealed a significant association between abdominal obesity and the incidence of symptomatic gallstones, which is generally consistent with our findings [35]. Previous research has used BMI as a predictor of the possibility of developing gallstones, yet the usefulness of this analysis is controversial. It is possible that BMI does not adequately reflect the occurrence of gallstones in obese people because the regional distribution of body fat, rather than the total quantity, is more crucial for the formation of gallstones [8]. WC is a measure of the degree of abdominal fat and a direct predictor of various metabolic disorders [36]. However, due to the strong association between WC and BMI, WC is limited as an independent predictor of BMI and does not differentiate between abdominal visceral fat and subcutaneous fat [27, 37, 38]. Therefore, an obesity index that accurately reflects the distribution of body fat and is easy to practice clinically becomes very important. Consistent with previous studies reporting negative effects associated with high WWI on fat metabolism [39], the authors also found a negative effect of WWI on gallstones risk. The present results support and provide more evidence for the link between central adiposity and gallstones in a general population.

The excessive accumulation of abdominal fat may be a key factor in the positive correlation between WWI and gallstones incidence. A majority of the cholesterol secreted in bile comes from hepatic synthesis, with a portion of it also coming through intestinal transit to the liver. The high levels of WWI may reflect the fact that the body is suffering from a burden of visceral adipose tissue, resulting in greater hepatic exposure to cholesterol and free fatty acids, and more bile cholesterol excretion. Visceral fat, but not subcutaneous fat, stimulates the development of insulin resistance, which raises the risk of forming gallstones, according to pathophysiologic mechanisms [10]. Higher plasma insulin levels, which occur as a result of insulin resistance, activate HMG-CoA reductase activity, and up-regulation of hepatocyte LDL receptors, leading to increased hepatic cholesterol secretion [35, 40]. Biddinger et al. found that hepatic insulin-resistant mice all developed gallstones, which may have been related to the up-regulation of the cholesterol transporters Abcg5 and Abcg8 [41]. A high serum leptin concentration reflects a high body fat percentage [42, 43], which contributes to gallstones development. According to a meta-analysis, people with gallstones have higher levels of leptin, which may point to a crucial role of central obesity in the development of gallstones [44]. Leptin promotes gallstones formation, which may be linked to decreased bile acid secretion via downregulation of the OB-Rb/AMPKα2/BSEP signaling pathway [45]. WWI is a more accurate measure of abdominal obesity, indicating central obesity independent of weight since it may combine the benefits of WC and lessen the association with BMI. It is clear that MRI and CT scan for assessing fat distribution are the most reliable, but also expensive and complex; therefore, considering the practicality, the use of WWI may be a more beneficial tool for evaluating the effects of visceral fat on gallstones. In the final adjusted model, WWI tertile 3 showed the higher prevalence of gallstones compared with tertile 1 (OR = 1.71, 95% CI:1.35‒2.17). Thus, overall, higher levels of WWI may lead to an increased risk of gallstones.

The level of WWI is positively correlated with the overall incidence of gallstones. We observed this relationship through a threshold effects analysis model, revealing that the incidence of gallstones approached its peak when WWI levels reached the inflection point (12.7), and then gradually declined as WWI levels increased. However, the sample size on the right side of the inflection point is small in proportion to the total sample size, which cannot significantly alter the overall positive correlation trend. As shown in Fig. 2, the relationship between WWI and gallstones presented an approximate, not quite symmetrical inverted U-shaped curve. It is noteworthy that over 96% of participants are located to the left of the inflection point, implying the reliability of the positive correlation between WWI and the incidence of gallstones. On the other hand, 243 participants on the right side presented a significant negative correlation. We conducted subgroup analysis to try to get a deeper understanding on what caused this inflection point. Taking gender as a stratification factor, we found that the fitted smoothing curves of females consistently lies atop that of males, indicating the presence of additional risk factors for gallstones in females. In the current age-stratified subgroup analysis, participants between the ages of 20 to 60 showed a significant positive association. Interestingly, the participants over the age of 60, as well as women, showed an inverted U-shaped curve similar to that of the total. The authors suggest that the reason for the inflection point is probably related to two factors: old age and gender. Generally, the difference between the sexes is attributed to the effect of hormones. Estrogen increases the excretion of cholesterol into the bile via upregulating the expression of the HMG-CoA gene, resulting in supersaturation of cholesterol in the bile [46, 47], which promotes gallstones formation. A study performed in rats showed that estrogen deficiency leaded to an impairment of the bile acid metabolic pathway, and the loss of the driving force of bile acids leaded to a decrease in biliary cholesterol excretion and accumulation of cholesterol in the liver [46, 48]. Estrogen is an important risk factor for gallstones [49], meanwhile, benefiting from estrogen-mediated reduction in visceral adiposity, premenopausal women were at lower risk of metabolic diseases than men [50]; however, the gradual decline in estrogen levels that follows menopause leads to a decrease in hepatic cholesterol excretion and a tendency to visceral fat accumulation, resulting in abdominal obesity and underlying metabolic abnormalities [51–53]. The estrogen has a significant impact on gallstones development since it regulates this process through hepatic lipid metabolism. We assumed that the reason for the inflection point may be related to the population of older women; nevertheless, more prospective researches need to be conducted to provide further evidence.

The incidence of gallstones varies between different populations, which may be due to environmental, cultural, and dietary factors [47, 54]. In our study, the positive association between WWI and gallstones prevalence was not significantly affected by other stratifications, including gender, diabetes, and sedentary time (all *P* for interaction > 0.05). Based on the fact that WWI is an effective method for evaluating the distribution of visceral adiposity, the results implied that the management of visceral adiposity may slow the process of gallstones formation. It is also important to note that rapid weight loss contributes to the formation of gallstones [55, 56]. As a result, we need to focus on ways to shed pounds such as personalized weight loss programs and dietary modifications [57].

There were, however, some limitations to this study. Firstly, the type of study was a cross-sectional analysis, thus causality could not be determined. Additionally, while accounting for several pertinent variables, it is hard to eliminate all the impact of confounding factors, so the conclusion should be given a cautious reading. Thirdly, because of the limited availability of survey items in the NHANES database, covariates were unable to include the medication use of participants, however, some medications (such as estrogen, progesterone and octreotide) predispose to gallstones [58]; as a result, we are unable to adjust the impact caused by medication use. Finally, the data for diagnosing gallstones were obtained from questionnaire results, potentially leading to recall bias. Given that most patients with gallstones are asymptomatic, the possibility of misclassifying individuals with or without gallstones exists. This could introduce bias into our results. Despite these limitations, there are some advantages to our study. This study provides a representation of the multiracial and diverse dietary adults in the U.S. as the sample from nationwide health surveys. Furthermore, the size of the sample allowed us to compare differences across populations in subgroup analyses.

## Conclusion

Our study found that higher WWI levels were connected with increased prevalence of gallstones. We realized that abdominal obesity and visceral adiposity reflect the burden of lipid metabolism, which is a condition that contributes to the formation of gallstones. The impact of abdominal obesity on the formation of gallstones may be related to hormone levels, which needs further research to confirm. These findings may facilitate the development of strategies to prevent gallstones through controlling visceral fat. However, more prospective studies are needed to validate our findings.

## Data Availability

All data in this study is available from NHANES database (www.cdc.gov/nchs/nhanes).
